# Primary appendiceal mucinous adenocarcinoma alongside with situs inversus totalis: a unique clinical case

**DOI:** 10.1186/1477-7819-8-49

**Published:** 2010-06-04

**Authors:** Athanasios Petrou, Alexandros Papalambros, Nikolaos Katsoulas, Konstantinos Bramis, Konstantinos Evangelou, Evaggelos Felekouras

**Affiliations:** 1 First Department of Surgery, National and Kapodistrian University of Athens Medical School, Laiko General Hospital, Athens, Greece; 2 Molecular Carcinogenesis Group, Department of Histology and Embryology, National and Kapodistrian University of Athens Medical School, Athens Greece

## Abstract

**Introduction:**

Mucinous adenocarcinoma is a rare neoplasm of the gastrointestinal tract and one of the three major histological subtypes of the primary appendiceal adenocarcinoma. The most common type of presentation is that of acute appendicitis and the diagnosis is usually occurred after appendectomy. The accurate preoperative diagnosis and management of the above condition represents a real challenge when uncommon anatomic anomalies such intestinal malrotation and situs inversus take place. Situs inversus totalis with an incidence of 0.01% is an uncommon condition caused by a single autosomal recessive gene of incomplete penetration in which the major visceral organs are mirrored from their normal positions.

**Case presentation:**

We present an unusual case of a 59 years old, previously healthy man presented with a left lower quadrant abdominal pain, accompanied with low fever, leukocytosis, anorexia and constipation. A chest radiograph demonstrated dextrocardia with a right side positioned stomach bubble. Both preoperative US and CT scan of the abdomen and pelvis declared situs inversus, with a characteristic thickening in its wall, appendix situated in the left lower quadrant of the abdomen. These findings reached to the diagnosis of acute appendicitis with situs inversus and a standard appendicectomy was performed. Pathologic evaluation established primary mucinous adenocarcinoma of the appendix and three months afterwards the patient underwent a subsequent extended left hemicolectomy.

**Conclusion:**

In conclusion, the occurrence of primary appendiceal mucinous adenocarcinoma along with situs inversus, definitely accounts as a unique clinical case. Even synchronous manifestation of primary mucinous adenocarcinoma of the appendix and situs inversus totalis represents an unusual anatomo-pathological entity, all physicians should be familiar having the knowledge to make an appropriate and accurate diagnosis that will lead to prompt and correct treatment.

## Introduction

Appendiceal carcinomas represent a relatively uncommon clinical entity. A recently published population-based study, concentrated in the period 1973-1998, from The National Cancer Institute's (NCI) Surveillance, Epidemiology, and End Results (SEER) program concluded that the age-adjusted incidence of appendiceal malignancies is approximately 0.12 cases per 1,000,000 people per year. The same study suggested the mucinous adenocarcinoma as the most frequent histologic type among the appendiceal identified carcinomas, while carcinoid was the second most frequent histologic diagnosis [1].

Mucinous adenocarcinoma represents an uncommon neoplasm of the gastrointestinal tract and one of the three major histological subtypes of the primary appendiceal adenocarcinoma [1,2] occupying only 0.01-0.2% [3] (while in some others reports the percentage reaches 0.4 to 1%) [3] of all gastrointestinal neoplasms. Appendiceal carcinomas are infrequently suspected preoperatively and, not as much of than 50% of cases are diagnosed during intraoperative exploration of the peritoneal cavity [4]. Diagnosis of mucinous adenocarcinoma of the appendix usually occurs after appendectomy or other explorative surgical procedures (appendiceal adenocarcinomas are noted to have a propensity for early perforation and peri-appendiceal abscesses) and consequent pathologic evaluation of the appendiceal specimen [3,5].

The formal right hemicolectomy is considered as the recommended treatment for all patients with nonmetastatic adenocarcinoma of the appendix. Though mucinous' adenocarcinoma spread to adjacent organs is detectable at presentation in a percentage 63%, the approximate overall 5-year survival rate at about 20.5%, arising to 55-60% after right hemicolectomy for nonmetastatic disease (widespread metastases are present in 10-50% [6] of the patients with appendiceal adenocarcinoma) and varying with stage and grade [5,6]. The use of appendicectomy alone in should be restricted for cases of adenocarcinoids limited to a small area of the appendix. To be more detailed, right hemicolectomy is considered to be the treatment of choice for all lesions with invasion beyond the mucosa, and, appendicectomy alone seems to be the ideal treatment for in situ and localised cases. This therapeutical option was supported by Varisco et al [7], on their meta-analysis (involving 100 patients), regarding the necessity of hemicolectomy in appendiceal carcinoma malignancies, with low tumour histology and no caecal involvement. As for mucinous appendiceal tumours with involvement of the appendiceal or distal ileocolic lymph nodes and documented peritoneal seeding or inadequate resection margin, authors suggest that there is no survival advantage in performing a right hemicolectomy alone [8], and, the application of intraperitoneal chemotherapy, in addition to the right hemicolectomy is recommended [9-14]. Aiming to be more precise, the current and recommended surgical/oncological management for patients diagnosed with peritoneal metastasis or peritoneal carcinomatosis, is consisted of the combined utilize of cytoreductive surgery and intraoperative/intra-abdominal chemotherapy [9-14]. The surgical procedure is directed to the visible disease removal throughout a thorough peritonectomy and visceral resections when is indicated. In order to avoid entrapment of tumour cells at operative sites and to destroy small residual mucinous tumour nodules, cytoreductive surgery is combined with intraperitoneal chemotherapy or to be more accurate intraperitoneal hyperthermic chemoperfusion. The latter includes 5-fluoro-2'-deoxyuridine (FUDR) plus leucovorin (LV) [15], 1000 mg/m^2 ^and 240 mg/m^2 ^respectively, either 200 mg/M^2 ^of oxaliplatin for a 2-h chemoperfusion [16], or combination of mytomicin-C (MMC) with either cisplatin [17] or fluorouracil [18]. Particularly intraperitoneal chemotherapy with mitomycin at 42 degrees C is a well tested chemotherapeutic agent. Fluorouracil is then given postoperatively for 5 days. If the mucinous neoplasm is minimally invasive and cytoreduction complete, these treatments result in a 20-year survival of 70% [14]. In the absence of a phase III study, this new combined treatment should be regarded as the standard of care for epithelial appendiceal malignant neoplasms and pseudomyxoma peritonei syndrome [14].

The rareness of the disease makes any therapeutic comprehensive investigation to seem mistrustful. Based on this motivation, formal studies have not been realised, and no evidence of a possible advantage of systemic chemotherapy for appendiceal adenocarcinoma exists. Based on the similarities between this type of appendix cancer and colon cancer, the treatment with systemic chemotherapy that is commonly used to treat colon cancer is also often used for the "colonic-type" appendiceal cancer. Some of the most commonly used intravenous chemotherapy agents consist of 5-FU, Leucovorin, Oxaliplatin and Irinotecan [9].

Avastin, a monoclonal antibody displaying an anti-angiogenic action, as well as bevacizumab are also sometimes added [19-21].

The location of the appendix in the left lower quadrant is extremely rare. Left-sided acute appendicitis and periappendiceal abscesses occur in association with two types of congenital anomaly: intestinal malrotation and situs inversus. Situs inversus totalis is an uncommon congenital anatomic abnormality in which the major visceral organs are reversed or mirrored from their normal positions. The above congenital condition affects all major structures within the thorax and the abdomen. Generally, the organs are simply transposed through the sagittal plane. The heart is located on the right side of the thorax, the stomach and spleen on the right side of the abdomen, while the liver and gall bladder on the left side. The left lung is trilobed, while the right lung bilobed, and moreover blood vessels, nerves, lymphatics and the intestines are also transposed. If the heart is swapped to the right side of the thorax, it is known as situs inversus with dextrocardia or situs inversus totalis [2,3].

This anatomic developmental anomaly totally differentiates our standard clinical differential diagnoses and complicates diagnosis of common intraperitoneal disease processes such as biliary colic, acute appendicitis and diverticulitis and the therapeutic management is often delayed as a result of the incompatible clinical finding [2-4].

It must be emphasized that up to 35% of the patients with appendiceal adenocarcinoma to have a second GI malignancy [6] that underlines the significant risk for both synchronous and metachronous neoplasms [1,22]. Such information is considered significant for the correct evaluation, diagnosis and management that will lead to the optimal surgical and oncological treatment.

Typically, patients with situs inversus have a normal life expectancy while the great majority of persons with situs inversus totalis are unconscious of their unusual anatomy, until they seek medical attention with plain chest erect film or ultrasonography for unrelated condition. Only in rare cases of situs inversus totalis with cardiac anomalies or for individuals with Kartagener syndrome and severe bronchiectasis, life expectancy is reduced, but always depended of the severity of the defect and the treatment efficiency [3,6,23].

## Case presentation

A 59 years old, previously healthy man, presented at the emergency room of First Dept. of Surgery, Athens Medical School, LAIKON GENERAL HOSPITAL, with a left lower quadrant abdominal pain, that arisen 24 h ago in the umbilical area, accompanied with low fever, as well as anorexia and constipation. Physical examination showed the patient to be febrile (body temperature of 38.4) with a mild suffering secondary to point tenderness on palpation of his left lower quadrant. Left-sided "Rebound" and "McBurney's" signs were also noted. Laboratory tests showed mild leukocytosis (11.6 × 109/L), accompanied with moderate polymorphonuclear predominance (89%) and an elevated CRP concentration (1 mg 55/L) and normal blood biochemistry analysis.

A standard preoperative chest radiograph demonstrated dextrocardia, with the stomach bubble situated on the right as well. Therewithal both Ultrasound (US) and Computed tomography (CT) abdominal scanning revealed situs inversus, with the appendix in the left lower quadrant of the abdomen, with remarkable thickening in its wall. It should be underlined that until then the patient had been unsuspicious of having situs inversus. These findings reached to the diagnosis of acute appendicitis with situs inversus and a standard appendicectomy with a McBurney - like (oblique) left lower quadrant muscle-splitting incision was performed. The patient recovered uneventfully and two days after was exited the hospital in a fine condition.

**Figure 1:**
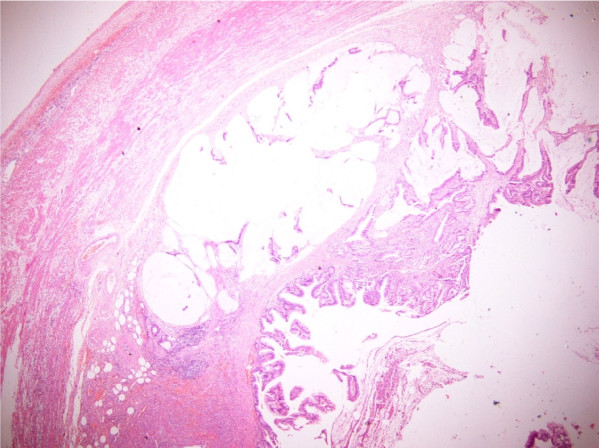
**Representative areas of the mucinous adenocarcinoma of the appendix (H&E counterstained, magnification: × 100)**.

**Figure 2:**
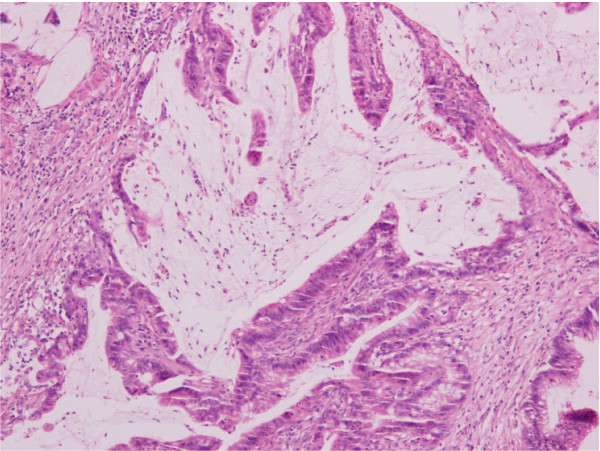
**Histological section (H&E counterstained) of the appendiceal mucous adenocarcinoma (magnification: ×200)**.

The appendix was submitted for histopathological examination. On gross examination accumulation of mucus within the lumen and focal thickening of the wall were observed. Histological examination revealed a mucinous adenocarcinoma of the appendix (Figure 1, 2) with the tumor to infiltrate the wall throughout the length of the muscular layer, with no invasion whatsoever of the subserosa and the periappendiceal fat. The mucinous appendiceal adenocarcinoma was classified as stage A on Duke's staging system and as T2N0Mx according to TNM classification. Additionally, there was no evidence of malignancy in the appendiceal stump and the patient was planned to a subsequent hemicolectomy.

**Figure 3:**
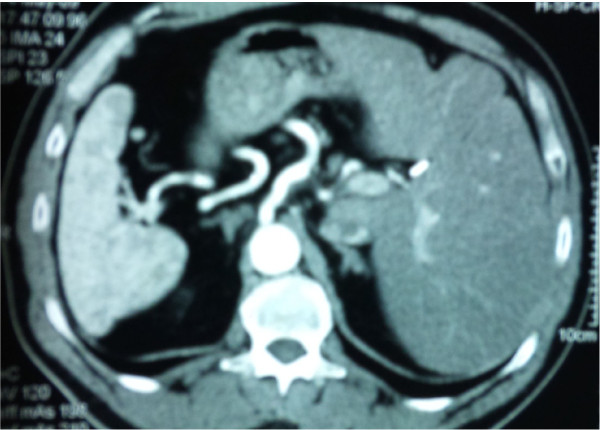
**Representative CT images of the abdomen one year after the operation during the patient's follow up**. The situs inversus totalis is clearly demonstrated.

**Figure 4:**
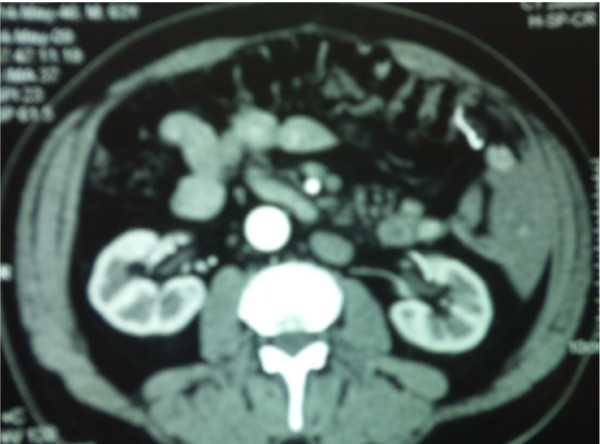
**Representative CT images of the abdomen one year after the operation during the patient's follow up**. The situs inversus totalis is clearly demonstrated.

Three months after the pathologic evaluation, the patient underwent a subsequent extended left hemicolectomy, in the pattern of the formal extended right hemicolectomy, with resection as well of 19 lymph nodes. On histology, no malignancy was indentified, only three lesions of tubular adenomas, while the lymph nodes presented plainly reactionary inflammation. No evidence of local recurrence or metastatic disease is confirmed by the standard follow-up, including yearly CT scanning of the abdomen (Figure 3, 4), and the patient is fit and in good spirit 16 months subsequent to the surgical treatment.

## Conclusions

Primary mucinous adenocarcinoma of the appendix constitutes a scarce malignancy of the appendix and often associated with a second GI malignancy of the gastrointestinal tract. Usually the appendiceal malignancies are mistaken for acute appendicitis and therefore their diagnosis follows appendectomy. Even the coexistence of primary appendiceal mucinous adenocarcinoma along with situs inversus totalis, definitely accounts as a unique clinical case, all physicians should be familiar having the knowledge to make an appropriate and accurate diagnosis that will lead to prompt and correct treatment.

## Consent statement

Written informed consent was obtained from the patient for publication of this case report and accompanying images. A copy of the written consent is available for review by the Editor-in Chief of this journal.

## Competing interests

The authors declare that they have no competing interests.

## Authors' contributions

AtP has edited the manuscript. AlP has operated and managed the patient. NK has edited the manuscript. KB has operated and managed the patient. KE has diagnosed and edited the manuscript and EF has supervised the whole attempt. All authors read and approved the final manuscript.
